# Urinary Bladder Perforation: A Forensic Case Report and Brief Literature Review

**DOI:** 10.7759/cureus.86831

**Published:** 2025-06-26

**Authors:** Athina Tousia, Evangelos Plantzas, Ioannis Platzas, Konstantinos Katsos, Dimitrios Kouzos, Ioannis Papoutsis, Dimitrios Vlachodimitropoulos, Nikos Goutas, Chara Spiliopoulou, Emmanouil I Sakelliadis

**Affiliations:** 1 Department of Forensic Medicine and Toxicology, National and Kapodistrian University of Athens School of Medicine, Athens, GRC

**Keywords:** autopsy, forensic medicine, forensic pathology, peritonitis, ulceration, urinary bladder

## Abstract

Urinary bladder ulceration and perforation are serious conditions that may develop due to various causes, most commonly trauma or complications from medical procedures. In rare instances, these conditions can occur without an identifiable cause. Bladder perforation can lead to peritonitis, a life-threatening complication with a reported mortality rate approaching 50%, primarily due to systemic inflammatory responses and multi-organ failure. Although spontaneous bladder rupture is uncommon, it has been associated with underlying conditions such as bladder outlet obstruction (BOO), malignancy, and chronic infections, or it may occur without a clearly identifiable cause (idiopathic). Prompt recognition and intervention are essential to improving patient outcomes.

We present the case of a 69-year-old woman with a medical history of diabetes mellitus, arterial hypertension, and knee osteoarthritis, for which she was receiving insulin, irbesartan, and hydrochlorothiazide. Additionally, approximately three years prior to her death, she was diagnosed with a urinary bladder neoplasm and underwent transurethral resection of the bladder tumor (TURBT), followed by intravesical therapy.

Postmortem examination (PME) revealed evidence of peritonitis. Histopathological examination confirmed the diagnosis of peritonitis, along with bladder ulceration and perforation. The cause of death (COD) was determined to be peritonitis secondary to bladder perforation.

## Introduction

Urinary bladder rupture or perforation is a rare but potentially fatal condition, with a reported mortality rate of up to approximately 50% due to its systemic complications [1]. The detrusor muscle, which forms part of the bladder wall, contributes to the organ’s structural integrity [2]. However, in certain circumstances, this durability may be compromised by various pathologies, resulting in bladder wall perforation or rupture.

Bladder rupture can occur due to blunt trauma, with a reported mortality rate of approximately 22%. Fortunately, such injuries are uncommon, as the bladder is well protected within the bony pelvis [3]. Trauma-related bladder rupture may be classified as extraperitoneal (urine leakage confined to the perivesical space) or intraperitoneal (urine leakage that disrupts the peritoneal surface) [4]. Iatrogenic rupture may also occur during or after surgical or endoscopic procedures, particularly in gynecologic or obstetric surgery, due to the close anatomical relationship between the female reproductive organs and the urinary tract [5]. In some cases, rupture may occur spontaneously [6], including as a late complication of radiotherapy [7].

Factors associated with spontaneous bladder rupture are summarized in Supplemental material 1, including bladder outlet obstruction (BOO), bladder malignancy, and idiopathic cases, among others [8].

In general, spontaneous bladder rupture is rare and challenging to diagnose. It is associated with considerable morbidity and mortality. To date, no specific guidelines exist for its investigation or management [9].

Peritonitis is a life-threatening condition defined as the inflammation of the peritoneum, caused by pathogens or non-pathogenic factors [10]. The pathophysiology of uroperitoneum (bladder rupture-induced peritonitis) involves a complex mechanism: urine entering the peritoneal cavity causes chemical irritation, which, if untreated, may lead to secondary bacterial infection [11]. The clinical presentation of uroperitoneum is often nonspecific and may mimic other abdominal emergencies, such as gastrointestinal perforations. Besides signs of acute abdomen, ascites have also been reported [12]. Because of its nonspecific presentation, diagnosis is frequently delayed, leading to challenges in treatment [13]. Computed tomography (CT) is considered the diagnostic gold standard in such cases [14]. Understanding the interplay between chemical irritation and subsequent infection is essential to guide effective treatment, which includes timely antibiotic therapy [15].

This paper presents a rare postmortem case of peritonitis due to bladder perforation caused by bladder ulceration, along with a brief review of relevant literature.

## Case presentation

We present the case of a 69-year-old woman referred to our department for a full medico-legal investigation. A postmortem examination (PME) was conducted, and tissue samples were collected for toxicological and histopathological analyses. 

A detailed summary of all macroscopic and histopathological findings is provided, along with the results of the toxicological examination.

A brief review of the literature was also performed. Relevant studies were identified through a comprehensive search of the PubMed database, using the terms “urinary bladder ulceration” and “urinary bladder perforation". There were no restrictions regarding publication date or geographic origin, and all included articles were in English. Studies citing the selected papers were also reviewed, and full texts were accessed when deemed relevant.

The patient, a 69-year-old Caucasian woman, was admitted to the emergency department (ED) with complaints of lower abdominal pain that had started approximately 10 days earlier. In the days prior to admission, she also experienced anorexia, malaise, and urinary retention. A Foley catheter was inserted, draining 800 cc of urine. Imaging studies, including chest radiography, lower abdominal ultrasound, and kidney, ureter, and bladder (KUB) radiography, showed no pathological findings. Subsequently, the patient developed dyspnea and loss of consciousness. Despite prompt intubation and cardiopulmonary resuscitation (CPR), resuscitative efforts were unsuccessful. A polymerase chain reaction (PCR) test for SARS-CoV-2 was positive. The body was referred for medico-legal autopsy, as the cause of death (COD) remained unclear.

According to information provided by the deceased’s relatives, her past medical history included diabetes mellitus, arterial hypertension, and knee osteoarthritis, for which she was receiving insulin, irbesartan, and hydrochlorothiazide. She had quit smoking three years prior and had a history of two vaginal deliveries. Notably, three years before her death, she was diagnosed with a urinary bladder neoplasm and underwent transurethral resection of the bladder tumor (TURBT). 

Nine months before her death, a small recurrent lesion was detected and removed through another TURBT. Three months prior to her passing, the patient underwent weekly intravesical therapy. Histopathological examination of the bladder tissue obtained during the TURBT showed acute and subacute inflammation, microabscess formation, and vascular congestion, with no evidence of malignancy.

Autopsy findings 

The body of the deceased underwent routine PME, which included the collection of tissue samples for histopathological analysis.

Aside from increased body weight (BMI > 27) and minimal medical interventions, no significant findings were noted during the external examination of the corpse.

On internal examination, signs consistent with attempted resuscitation were evident, including rib and sternal fractures. The brain showed no gross abnormalities. The lungs exhibited mild congestion and edema, along with localized vascular inflammation. The heart was enlarged, with features of cardiomyopathy and ischemic injury. Significant coronary artery disease was present, with Va-Vb-type atheromatous lesions [16] and marked narrowing of both major coronary arteries. The abdominal aorta showed advanced atheromatosis with calcifications.

In the abdominal cavity, adhesions between the sigmoid colon and bladder were observed. Approximately 100 mL of purulent exudate was noted in the lower peritoneum and the rectouterine pouch, indicative of localized infection or inflammation. The esophagus, liver, and pancreas appeared normal. The gallbladder was distended, and calcifications were found on the splenic capsule, likely sequelae of past inflammation. Bile-like fluid was present in the stomach.

The kidneys had a micronodular surface, consistent with chronic arterial hypertension, and showed architectural disruption. The bladder mucosa appeared reddened and thickened, while the bladder cavity was filled with hemorrhagic fluid. 

Macroscopic examination of the internal genitalia revealed adhesions between the bladder and the uterine body. No perforation was macroscopically evident. As expected for the patient's age, the internal genitalia exhibited atrophic changes. Histologically, the endometrium was atrophic, and the adnexa (fallopian tubes and ovaries) showed no specific pathological changes, except for mild-to-moderate inflammatory infiltrates in the perifallopian tissue. 

**Figure 1:**
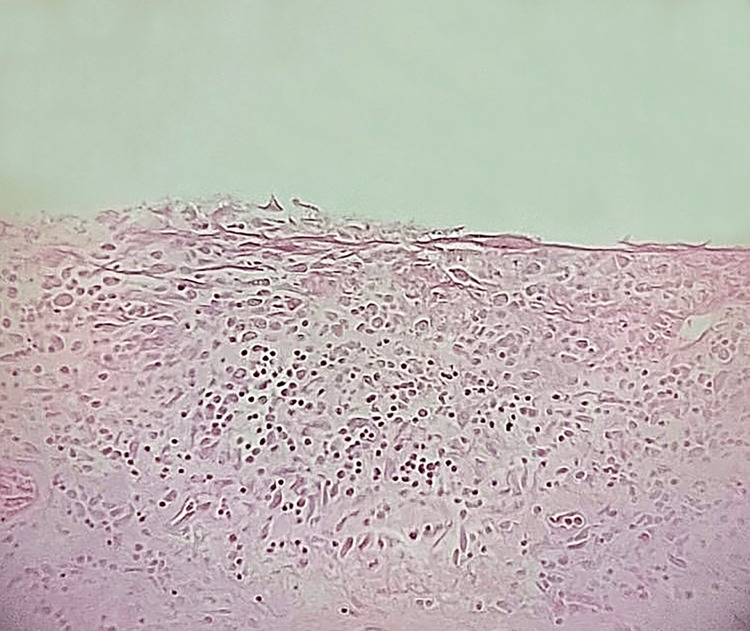
Severe inflammatory lesions and thickening of the colonic serosa. A population of macrophages, lymphocytes, and fibroblasts is observed (H&E X100 magnification)

**Figure 2:**
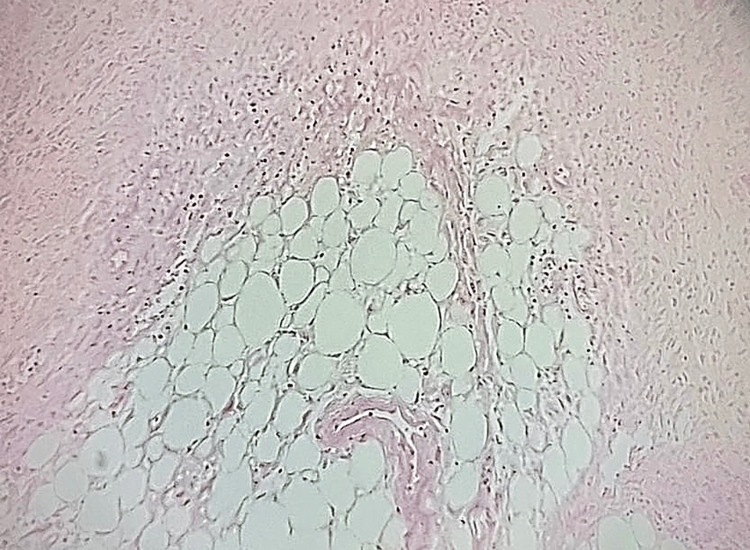
Dense inflammatory infiltration with exudations and necrosis of the adipose tissue consistent with peritonitis (H&E X50 magnification)

As noted, histopathological examination confirmed peritonitis. The sigmoid colon displayed dense inflammatory infiltrates composed of a mixed population of immune cells, affecting the serosa and adjacent adipose tissue, with several foci of liponecrosis. Importantly, no inflammatory cells were identified in the mucosa, submucosa, or muscularis propria, suggesting no evidence of concurrent diverticulitis. These findings are illustrated in Figure 1 and Figure 2.

**Figure 3:**
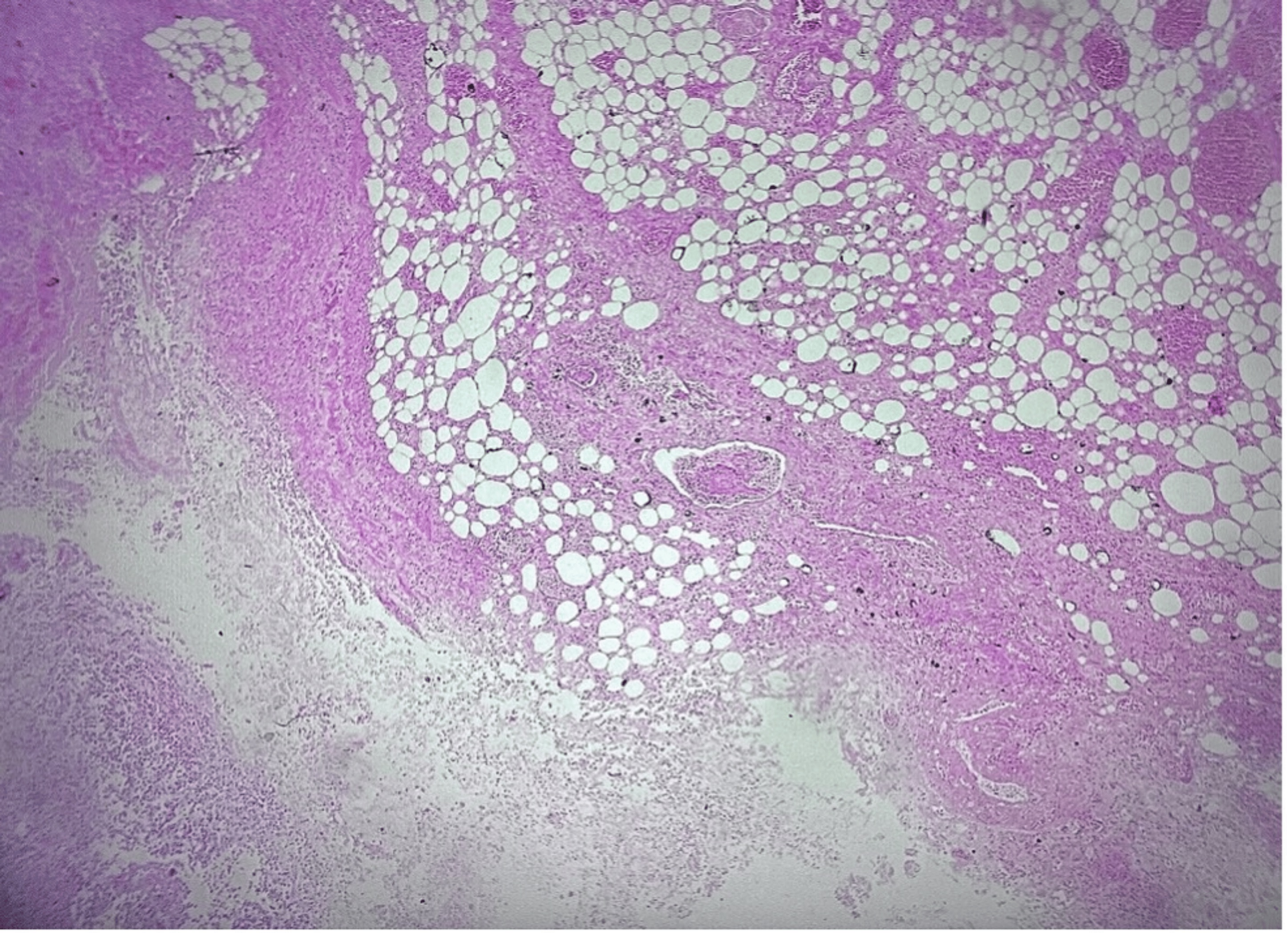
Perforation of the urinary bladder wall (H&E X25 magnification)

**Figure 4:**
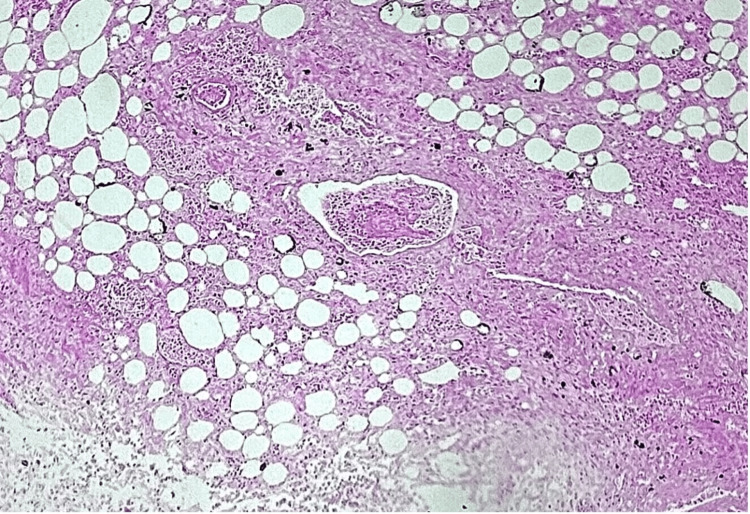
Extension of the inflammatory infiltrate to the adipose tissue. Small vessels with thrombus in the lumen (H&E X25 magnification; zoomed-in as compared to Figure 3)

Histologically, the urinary bladder showed extensive mucosal ulceration, with transmural extension of the inflammatory infiltrates into the adjacent adipose tissue. These findings are illustrated in Figure 3 and Figure 4. 

The toxicological examination did not reveal any significant findings. 

## Discussion

Urinary bladder perforation can occur due to a variety of etiologies, including iatrogenic causes, trauma, neoplastic processes, and infections. In this specific case, the patient had a prior diagnosis of urinary bladder malignancy. While bladder malignancies are a rare cause of spontaneous rupture leading to peritonitis [17], no evidence of active neoplasia was found during the PME or subsequent histopathological analysis. 

According to the patient’s medical history, three years prior to her death, she underwent TURBT for the excision of the bladder neoplasm. Bladder ulceration following TURBT may occur due to surgical trauma to the bladder lining, which creates areas vulnerable to ulcer formation [18]. 

Histopathological findings in this case also indicated chronic inflammation. Cystitis, or inflammation of the bladder, can lead to rupture and peritonitis and may be caused by several factors, including bacterial infections [19,20], interstitial cystitis [21], and xanthogranulomatous cystitis [22]. Other contributing factors include radiation therapy [23] and certain medications [24]. 

In interstitial cystitis, the development of mucosal ulcers (Hunner’s ulcers) can occur. These lesions are sometimes treated with transurethral resection (TUR), a procedure that carries a risk of perforation, especially when performed on areas already thinned by ulceration [25].

Additionally, the patient had undergone Foley catheterization multiple times over the years, including during her final hospitalization. Chronic catheter use has been associated with an increased risk of bladder perforation [26].

Prolonged urinary retention is another recognized risk factor for spontaneous bladder rupture. Overdistension of the bladder can cause hypoxia and ischemia of the bladder wall, creating conditions favorable for ulceration, perforation, and subsequent peritonitis [27]. Of note, the patient tested positive for COVID-19 during hospitalization. Emerging evidence suggests that SARS-CoV-2 infection may affect the urinary system, with reports of COVID-19-associated cystitis (CAC). This condition is hypothesized to result from increased urinary excretion or local expression of inflammatory cytokines [28]. 

Furthermore, based on information provided by family members, the patient had a history of diabetes mellitus, a condition associated with diabetic cystopathy [29]. This complication involves impaired bladder sensation and contractility, increased residual urine volumes, and bladder wall distension due to autonomic neuropathy. These changes may increase the bladder’s susceptibility to ulceration and rupture, especially in the presence of infection or other stressors [30].

Other causes of non-traumatic bladder perforation reported in the literature include radiation exposure [31], pregnancy [32], and heavy alcohol consumption [33].

## Conclusions

This case describes a rare but ultimately fatal instance of peritonitis resulting from bladder wall ulceration and subsequent perforation. From a clinical point of view, it is crucial to emphasize the importance of prompt and appropriate imaging, such as CT scanning, which can significantly aid in early diagnosis and intervention. In this case, the PME revealed that the ulceration had progressed to a full-thickness perforation, allowing urine to leak into the peritoneal cavity, thereby triggering a severe systemic inflammatory response. Notably, classic signs such as overt uroperitoneum were absent, underscoring the importance of meticulous macroscopic examination in identifying less obvious causes of peritonitis. In complex or unclear cases, detailed anatomical assessment during the PME is essential, especially when the clinical history or initial findings do not point to a clear COD.

Histopathological examination played a pivotal role in this investigation, confirming the presence of mucosal ulceration and establishing the source of peritonitis. This underscores the importance of histology in forensic pathology, not only as a confirmatory measure but also as a critical diagnostic tool in uncovering subtle yet fatal pathological processes. Forensic pathologists should remain vigilant for the possibility of bladder perforation, particularly in patients with known risk factors such as chronic bladder inflammation, malignancy, or prolonged catheter use. A comprehensive medical history, obtained before autopsy, can provide essential context and guide a more focused and effective investigative approach. Ultimately, this case reinforces the need for early recognition and appropriate management of urinary tract pathology to prevent such devastating outcomes.

## Appendices

Supplemental material 1 

**Table 1:** Factors associated with spontaneous bladder rupture BOO: bladder outlet obstruction, NVD: natural vaginal delivery, IAP: intra-abdominal pressure, TCC: transitional cell carcinoma, SCC: squamous cell bladder carcinoma. Adapted From: [8].

Factors associated with spontaneous bladder rupture	
BOO	Intravesical: vesicolith, bladder neck papilloma. Extravesical: urological, non-urological
Pregnancy	Post-NVD, intrapartum, other
Infections	Cystitis (uncertain/unspecified origin), tuberculosis, fungal, parasitic, bacterial (non-tuberculosis), gangrenous/necrotizing, eosinophilic, granulomatous (non-tuberculosis), viral, interstitial
Overdistention	Drug-associated: alcohol, anticholinergic, benzodiazepine, opioid, drug not specified. Non-drug-associated: neurogenic bladder
Increased IAP	Vomiting, seizures
Bladder malignancy	Malignancy-associated: radiation-associated fibrosis (pelvic malignancies of non-bladder origin), bladder TCC, bladder SCC, bladder unspecified type, bladder leiomyosarcoma
Bladder pathology	Non-malignancy-associated: bladder diverticulum, bladder amyloidosis, fatty infiltration of the bladder
Other	Warfarin toxicity, hemophilia
Not identified	Idiopathy
